# The Cellular Genesis of Metabolic Syndrome and the Role of Anti-urate Drugs in Hyperuricemia Patients: A Systematic Review

**DOI:** 10.7759/cureus.62472

**Published:** 2024-06-16

**Authors:** Maujid Masood Malik, Nency Ganatra, Rosemary Siby, Sanjay Kumar, Sara Khan, Srilakshmi K Jayaprakasan, Doju Cheriachan, Heet N Desai, Leslie Sangurima

**Affiliations:** 1 Biomedical Sciences, King Faisal University, Al-Ahsa, SAU; 2 Internal Medicine, California Institute of Behavioral Neurosciences and Psychology, Fairfield, USA; 3 Internal Medicine, Bahria University Medical and Dental College, Pakistan Navy Ship (PNS) Shifa Hospital, Karachi, PAK; 4 Pediatrics, Dr. Bhim Rao Ambedkar Medical College and Hospital, Bengaluru, IND; 5 Emergency Medicine, California Institute of Behavioral Neurosciences and Psychology, Fairfield, USA

**Keywords:** xanthine oxide, kidneys, xor inhibitors, inflammation, xanthine oxidoreductase (xor), oxidative stress, hyperuricemia, metabolic syndrome

## Abstract

Hyperuricemia results due to the underexcretion of uric acid through kidneys or overproduction due to either intake of purine-rich foods, a high caloric diet, or a decreased activity of purine recycler hypoxanthine-guanine phosphoribosyl transferase (HGPRT). Increased xanthine oxidoreductase (XOR) enzyme activity may contribute to hyperuricemia. Literature provides growing evidence that an independent component that contributes to the development of metabolic syndrome (MetS) and associated comorbidities is hyperuricemia. Thus, precise cellular mechanisms involved during MetS and related comorbidities in hyperuricemia, and the role of anti-urate medicines in these mechanisms require further investigations. We searched online libraries PubMed and Google Scholar for data collection. We used Preferred Reporting Items for Systematic Reviews and Meta-Analyses (PRISMA) 2020 guidelines for literature identification, selection, screening, and determining eligibility to produce unbiased meaningful outcomes. We applied quality assessment tools for the quality appraisal of the studies. And, outcomes were extracted from the selected studies, which revealed the relationship between hyperuricemia and MetS components by causing inflammation, endothelial dysfunction, oxidative stress, and endoplasmic reticulum stress. The selected studies reflected the role of xanthine oxide (XO) inhibitors beyond inhibition. This systematic review concluded that hyperuricemia independently causes inflammation, oxidative stress, endothelial damage, and endoplasmic reticulum stress in patients with hyperuricemia. These mechanisms provide a cellular basis for metabolic syndrome and related comorbidities. In this context, XO inhibitors and their beneficial effects go beyond XOR inhibition to ameliorate these pathological mechanisms.

## Introduction and background

Uric acid (UA) is an organic compound. According to the National Health and Nutrition Examination Survey (NHANES) conducted between 2007 and 2016 in the United States of America, hyperuricemia was more common in 2007-2008 (19.1%) than it was between 1988 and 1994 (21.5%). Later, this increase was noticed continuously every year. There was a prevalence of 20.2% in males while in females it was 20.0% according to the 2015-2016 report [1-3].

Humans and other Hominidae primates have some strategies for controlling hyperuricemia. The first is an increase in the uric acid elimination through its transporters and the second is an increased activity of the purine recycler hypoxanthine-guanine phosphoribosyl transferase (HGPRT) [4]. Some urate transporters eliminate one-third of UA from the digestive system, while the remaining two-thirds are eliminated through the kidneys. Urate production and reabsorption in the renal and digestive system are connected to human sodium phosphate cotransporter types (NPT1-4), organic anion transporters (OAT1-4), urate transporter (URAT1), glucose transporters (GLUT9), and adenosine triphosphate (ATP) binding cassette subfamily member (ABCG2), and play a contributory part in hyperuricemia.

Hyperuricemia also occurs due to decreased action of mutated HGPRT that changes hypoxanthine to inosine monophosphate (IMP) and guanosine to guanosine monophosphate (GMP); these disorders are called Lesch-Nyhan syndrome and Kelley-Seegmiller syndrome, respectively [5,6]. After the under-excretion of UA through kidneys and the decreased activity of HGPRT, another reason for the increased OA owes to foods like purine-rich foods, meat, or shellfish which are among the common risk factors in addition to genetic variables for hyperuricemia and gout. Likewise, other risk factors are alcohol, especially beer, and foods high in sugar, particularly fructose, which produces purine when consumed. The glycolysis cascade phosphorylates the fructose to fructose-1-phosphate consuming adenosine triphosphate (ATP). So, excess fructose consumption causes the body to use a lot of ATP, produce a lot of adenosine diphosphate (ADP) and adenosine monophosphate (AMP), and in turn increases the activity of the enzyme AMP deaminase, which converts AMP into IMP. This phenomenon accelerates the formation of UA [7].

Binary function of urates

UA controls 50% of the antioxidant activity in serum and is involved in the processes that scavenge approximately 70% of all human blood oxidants. UA demonstrates antioxidant properties and this strong antioxidant property may shield endothelium from reactive oxygen species (ROS), formed extracellularly at physiological concentrations, and scavenges peroxyl radicals like peroxynitrite (ONOO-) and carbon-centered radicals in plasma. UA can combine with peroxynitrite to generate uric acid derivatives like nitration/nitrosation, which can release nitric oxide (NO) and boost the effective proportion of NO in blood. However, when compared with other oxidants, UA produces radicals that primarily target membranes, low-density lipoprotein (LDL) cholesterol, and lipids [8].

The hyperuricemic status builds up oxidative stress when the ROS are formed by increased activity of xanthine oxidase during the UA metabolism. In such an environment, nicotinamide adenine dinucleotide phosphate (NADPH) expression increases, and damage to the mitochondria occurs, resulting in mitochondrial ROS (mtROS) production. ROS activates inflammasome, nucleotide-binding domain NOD-like receptor protein 3 (NLRP3) receptors that activate inflammatory signaling pathways producing inflammatory cytokines.

Another pro-atherogenic effect of ROS is the oxidation of an anti-atherogenic factor, NO, that regulates the tone of the vessels. UA also causes a decrease in the formation of NO through the high-mobility group box-1 protein-receptor for advanced glycation end products (HMGB1-RAGE) pathway, where increased HMGB1 synthesis and RAGE expression result in the removal of phosphate from endothelial nitric oxide synthase (eNOs), which reduces the formation of NO. Additionally, UA itself may cause the production of inflammatory cytokines, adhesion molecules, and chemokines, and influence cell proliferation and death of human endothelium. Besides, UA locally triggers the renin-angiotensin system (RAS), forming angiotensin II and increasing oxidative stress within the cell.

In a nutshell, the oxidative nature of UA in hyperuricemia accelerates the development of numerous illnesses [4]. A strong association exists between MetS and comorbidities, such as pro-inflammatory and prothrombotic states, and microalbuminuria caused by hyperuricemia. MetS relates to a group of factors like central obesity and dyslipidemia, which is characterized by high triglyceride levels and decreased high-density lipoprotein (HDL)-cholesterol, hypertension, and increased fasting blood glucose (FBG) [9]. In total, 20-25% of adults all over the world suffer from MetS. These people have a two-fold greater risk of dying from cardiovascular disease (CVD) and a three-fold increased risk of coronary heart disease (CHD) and stroke as compared to those who do not have MetS [10,11].

The diagnosis of MetS is based on the recommendations of the International Diabetes Federation (IDF) [12,13] and revised National Cholesterol Education Programs Adult Treatment Panel III (NCEP ATP III) criteria (Table 1) [14].

**Table 1:** Diagnostic criteria for metabolic syndrome. *A person will be deemed to have metabolic syndrome if two out of the following four variables are deranged along with the WC. WC: waist circumference; IDF: International Diabetes Federation; TGs: triglycerides; HDL-C: high-density lipoprotein cholesterol; BP: blood pressure

Variables	IDF criteria*
WC	Raised WC, males ≥90 cm, females ≥80 cm
Fasting plasma glucose	≥5.6 mmol/L or known diabetic/taking diabetes mellitus therapy
Hypertension	Systolic BP≥130 mmHg, diastolic BP≥85 mmHg/diagnosed hypertensive/on therapy
HDL-C	Men (<1.03 mmol/L), women (<1.29 mmol/L) or on HDL enhancing therapy
TGs	≥1.7 mmol/L/taking TG lowering drugs

The NCEP ATP III criteria were updated by the American Heart Association and the National Heart, Lung, and Blood Institute, supported its general applicability and validity, and suggested its use with little adjustments and clarifications [15]. The diagnostic criteria of the MetS defined by the revised NCEP ATP III minutely differs from the IDF criteria. According to the revised NCEP ATP III criteria, MetS is diagnosed when the patients satisfy three variables out of the five mentioned in Table 1.

Due to the encouraging results of xanthine oxidase (XO) inhibitors on clinical outcomes in hyperuricemic individuals, more focus has been placed on allopurinol and febuxostat (FBX), which are commonly used as urate-lowering drugs. Both inhibit the XO while adopting a different pathway from each other. It is noticed that allopurinol-treated gout patients have lower rates of cardiac and vascular ailments and mortalities. Also, the possibility of manic issues, chronic renal disorders, and carcinoma of the prostate decreases. These results demonstrate that allopurinol has some positive effects in terms of antioxidant and anti-inflammatory activities besides decreasing urate levels. Likewise, a selective xanthine oxidase inhibitor, febuxostat also shows biological effects like antioxidant, anti-inflammatory, and anti-apoptotic [16-18].

This systematic review intended to probe the precise cellular mechanisms responsible for initiating and forming the various MetS constituents in hyperuricemia. We also intended to investigate the extent of the influence and role of commonly prescribed anti-urate medicines, as the antioxidants and anti-inflammatory mediators, against the comorbidities associated with hyperuricemia-initiated MetS and its components.

## Review

Preferred Reporting Items for Systematic Reviews and Meta-Analyses (PRISMA) 2020 guidelines were followed [19]. An online search was conducted in PubMed and Google Scholar to extract the data. We created keywords using the building block technique, the Boolean operators, and the Medical Subject Heading (MeSH) from PubMed's MeSH database to perform an extensive literature search.

Keywords created using the building block technique

The keywords include combinations of words using the building block technique, encompassed metabolic syndrome and hyperuricemia; Lesch-Nyhan syndrome; gout and oxidative stress; reactive oxygen species; free radicals; oxidants; endoplasmic reticulum stress; febuxostat; and allopurinol.

MeSH created in PubMed MeSH database

The keywords, including the combination of words created using PubMed MeSH, included metabolic syndrome and etiology; metabolic syndrome and genetics; metabolic syndrome and physiopathology; metabolic syndrome and etiology; metabolic syndrome and genetics; metabolic syndrome and physiopathology; hyperuricemia; Lesch-Nyhan syndrome; hyperuricemia and etiology; hyperuricemia and metabolism; hyperuricemia and physiopathology; Lesch-Nyhan syndrome and metabolism; Lesch-Nyhan syndrome and physiopathology; gout and etiology; gout and metabolism; gout and physiopathology; oxidative stress and etiology; oxidative stress and physiology; endoplasmic reticulum stress and drug effects; endoplasmic reticulum stress and etiology; endoplasmic reticulum stress and pathophysiology; oxidative stress and drug effects; allopurinol and therapeutic use; and febuxostat and therapeutic use.

Keywords created using Boolean operators based on relevance

The keywords, including the combination of words created using Boolean operators based on relevance, included metabolic syndrome and hyperuricemia; Lesch Nyhan syndrome; gout; metabolic syndrome and febuxostat; metabolic syndrome and allopurinol; oxidative stress, reactive oxygen species, free radicals, oxidants, and febuxostat; oxidative stress, reactive oxygen species, free radicals, oxidants and allopurinol; endoplasmic reticulum stress and febuxostat; and endoplasmic reticulum stress and allopurinol. A combination of keywords, created using the building block technique and the MeSH keywords, for PubMed search was used to extract the relevant studies (Table 2).

**Table 2:** The number of studies identified after the database search. MeSH: medical subject heading

Search by MeSH + regular keywords (combination) using building block technique, advance technique, and Boolean technique	PubMed	Google Scholar
Search by MeSH and regular keywords (combination)	1239	Not done
Search by using all regular keywords	01	07
Metabolic syndrome and hyperuricemia, Lesch Nyhan syndrome, gout	23706	13400
Metabolic syndrome and febuxostat	50	4940
Metabolic syndrome and allopurinol	189	34100
Oxidative stress, reactive oxygen species, free radicals, oxidants, febuxostat	175	1390
Oxidative stress, reactive oxygen species, free radicals, oxidants, allopurinol	2280	18500
Endoplasmic reticulum stress and febuxostat	05	564
Endoplasmic reticulum stress and allopurinol	12	5010

Inclusion and exclusion criteria

Studies involving adults of both sexes, studies published in the last five years, articles published in English, studies on human beings, cross-sectional/case-control/cohort studies, randomized control trial (RCT) studies, and reviews including systematic reviews and meta-analyses were included. The study excluded editorials, letters, case reports, case series, grey literature, non-peer-reviewed articles, animal studies, irrelevant papers, and studies on subjects <18 years old.

Study selection/screening

**Figure 1:**
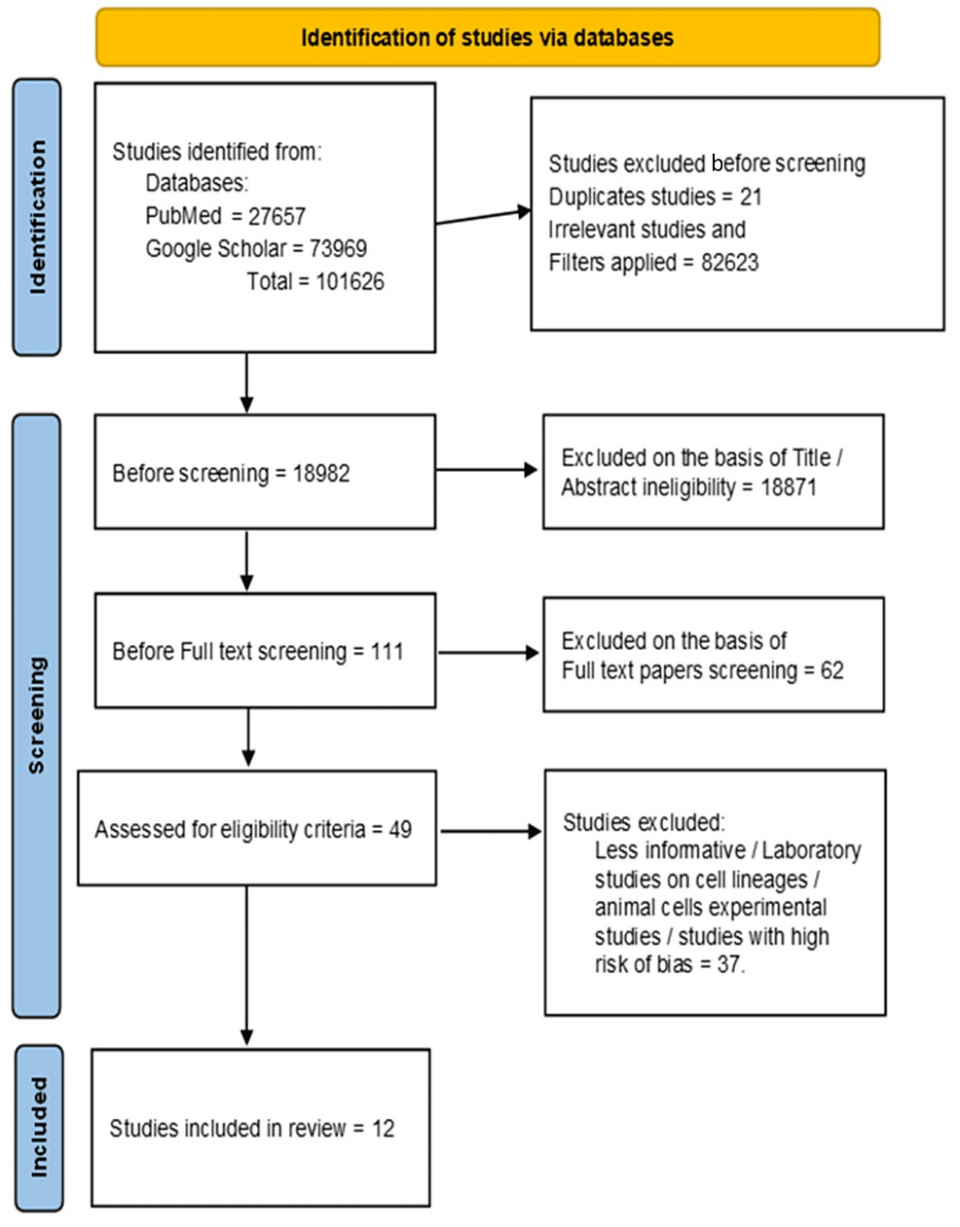
PRISMA 2020 flow diagram depicting study selection. PRISMA: Preferred Reporting Items for Systematic Reviews and Meta-Analyses

The Preferred Reporting Items for Systematic Reviews and Meta-Analyses (PRISMA) 2020 guidelines were used in the literature identification, selection, screening, and determining eligibility (Figure 1) [19].

Two authors (Malik and Ganatra) independently identified and recognized the acceptability of the papers. Based on inclusion and exclusion criteria, they determined the appropriateness of the documents with consensus. The third author (Siby) resolved the difference of opinion issues, if any. Two authors performed an eligibility assessment to produce precise, accurate, unbiased, transparent, and meaningful results. Quality assessment was accomplished by the two authors (Malik and Ganatra) using various appraisal tools meant for included study designs. Studies with low bias were included in the review except for an RCT revealing attrition bias.

Results

Twelve articles were selected and their outcomes were elucidated. An observational cohort/updated meta-analysis and two cross-sectional studies revealed a positive role of hyperuricemia in the manifestation of MetS. One systematic review, a meta-analysis, and three narrative reviews observed that hyperuricemia may cause insulin resistance, oxidative stress, endothelial dysfunction, and inflammation which form the basis for MetS, and XO inhibitors are effective. Two other narrative reviews concluded that xanthine oxidoreductase (XOR) is the main culprit causing different elements of MetS and XO should be the therapeutic target. However, one narrative review identified UA as a sign of oxidative stress and XO inhibitors responsible for endothelial dysfunction because it caused a decrease in the production of NO. One observational cohort study showed the positive effect of febuxostat on microalbuminuria in gout patients and another multicentric RCT considered febuxostat a more effective drug than allopurinol in heart failure patients with hyperuricemia (Table 3).

**Table 3:** Observations from the studies included in this review. UA: uric acid; MetS: metabolic syndrome; NLRP3: NOD-like receptor family pyrin domain containing 3; AMPK-mTOR-mROS: adenosine monophosphate (AMP)-activated protein kinase (AMPK)-mammalian target of rapamycin (mTOR)-mitochondria-derived reactive oxygen species (mROS); HIF-1α: hypoxia-inducible factor 1-alpha; ROS-IRS1/Akt: reactive oxygen species-insulin receptor substrate-1 (IRS1)/serine or threonine kinase or protein kinase B; XO: xanthine oxide; NO: nitric oxide; XOR: xanthine oxidoreductase; NAFLD: nonalcoholic fatty liver disease; NA: not applicable

Studies	Country	Study design	Sample size	Conclusion/outcomes	ROB
Huang et al. 2020 [20]	China	Cross-sectional study	1035	MetS and its elements are substantially related to hyperuricemia, particularly in elderly people, and should be monitored closely in old and middle-aged people, particularly in females	Low risk of bias
Chen et al. 2021 [21]	Australia	Observational cohort study/updated meta-analysis	3779	UA enhances the risk of developing MetS and in middle and old age people UA should be closely watched	Low risk of bias
Yao et al. 2022 [22]	Tibet	Cross-sectional study	307	UA levels are notably related to metabolic syndrome and its associated elements irrespective of age and sex	Low risk of bias
Alem 2018 [23]	Saudi Arabia	Systematic review meta-analysis	NA	Existing knowledge holds that oxidative stress and impaired endothelial activity are responsible for heart failure. Allopurinol may be a good drug to use in hyperuricemia, because of its antioxidant properties	Low risk of bias
Joosten et al. 2020 [24]	United States of America	Narrative review	NA	This study identifies asymptomatic hyperuricemia’s role in causing inflammation through innate immunity and activating epigenetic cascades. Drugs affecting these inflammation processes along with the UA-lowering drugs may benefit in decreasing inflammation and its disease effects	Low risk of bias
Yu and Cheng 2020 [25]	China	Narrative review	NA	This review discussed UA’s role in causing arterial stiffness and inflammation which is NLRP3 inflammasome dependent and is activated through AMPK-mTOR-mROS and HIF-1α. Cardiomyocytes with hyperuricemia exhibit insulin resistance due to the activation of the ROS-IRS1/Akt phosphorylation pathway and are positively correlated with the various components of MetS. Moreover, febuxostat effectively improves cardiovascular events as compared to allopurinol	Low risk of bias
Gherghina et al. 2022 [26]	Romania	Narrative review	NA	In hyperuricemia, the oxidative stress and inflammatory processes are responsible for UA-related pathogenesis. This could be because of UA formation pathways and intracellular reactions of UA. Moreover, XO inhibitors have a better effect on endothelial function as compared to uricosuric drugs	Low risk of bias
Battelli et al. 2018 [27]	Italy	Narrative review	NA	It emphasizes that pathology of the various components of metabolic syndrome involves XOR which affects the regulative function of NO production, fat accumulation, ROS production, and uric acid production. Uric acid subsequently causes the development of not only insulin resistance, increased blood triglycerides, obesity, blood pressure, and renal disease but also NAFLD. The study states that the XOR inhibitors use should be confined to patients with hyperuricemic effects because it plays a useful role in redox equilibrium. Prospective studies on Japanese and Italian residents advocate that hyperuricemia should be watched closely especially in elders if they have impaired blood glucose, high triglycerides levels, and a prehypertension scenario	Low risk of bias
Si et al. 2021 [28]	China	Narrative review	NA	The main culprits of heart failure in hyperuricemia patients are XO and ROS production, so XO should be the therapeutic target in such patients	Low risk of bias
Packer 2020 [29]	United Kingdom	Narrative review	NA	UA is a sign of oxidative stress and it is not dangerous rather drugs used to lower it cause the decreased production of NO	Low risk of bias
Vaidya et al. 2020 [30]	Nepal	Observational cohort study	114	The gout patient’s microalbuminuria was decreased by the febuxostat	Low risk of bias
Suzuki et al. 2021 [31]	Japan	Multicenter RCT	263	Febuxostat proved more effective therapeutically than allopurinol in heart failure patients with raised uric acid	High risk of bias/ attrition

Discussion

UA is a weak acid under physiologic conditions. After originating from purines, UA circulates as a soluble deprotonated urate anion in the blood. When this ionized form of UA is increasingly circulating in the blood, it combines with sodium ions to form the needle-shaped crystal called monosodium urate (MSU) [32]. Under pathological conditions, increased levels of UA represent hyperuricemia. Hyperuricemia is characterized by increased urate levels exceeding the normal concentrations of less than or equal to 7 mg/dL in males and 6 mg/dL in females. In the absence of inflammation, high concentrations of UA represent asymptomatic hyperuricemia. It is evident from the literature review that hyperuricemia is linked with type 2 diabetes mellitus (T2DM), obesity, insulin resistance, dyslipidemia, chronic kidney disease (CKD), nonalcoholic fatty liver disease (NAFLD), cardiovascular illnesses, and MetS [33,34].

In the past decade, sufficient information has been available in the literature that advocates a strong association between hyperuricemia and MetS components. Huang et al., in their study, found a noticeable relationship between the MetS elements and hyperuricemia, particularly in elderly people, and should be monitored closely in old and middle-aged people and particularly in females [20]. Chen et al. concluded that UA is positively related to the possibility of having metabolic syndrome, and UA in middle and old age people should be closely watched [21]. Likewise, a study conducted by Yao et al. identified the association between high levels of UA and the various elements of MetS, irrespective of age and sex [22].

According to the IDF and revised NCEP ATP III criteria, waist circumference (obesity), hypertriglyceridemia, low HDL-C (dyslipidemia), insulin resistance (T2DM), and high blood pressure (endothelial dysfunction, arterial stiffness) are the various components of MetS. Yet there are additional comorbidities as well, including prothrombotic and inflammatory conditions, non-alcoholic fatty liver disease, and microalbuminuria [9,12-14].

Many studies have provided useful data about the cellular genesis of the various elements of MetS in hyperuricemia patients. Studies precisely and convincingly reveal that these cellular changes directly result from mechanisms like inflammation, oxidative stress, endoplasmic reticulum stress, and endothelial damage, initiated by hyperuricemia. They also mentioned that hyperuricemia is an independent mechanism that causes these alterations.

According to Alem, it was noticed that oxidative stress and impaired endothelial activity are responsible for heart failure [23]. A study by Joosten et al. identified asymptomatic hyperuricemia and its role in causing inflammation through innate immunity and activating epigenetic cascades. It has also been suggested that UA may impact mental capacity, drive, reaction time, and impulsivity [24]. Another report by Gherghina et al. concluded that in hyperuricemia the oxidative stress and inflammatory processes, actually originate as a result of UA formation pathways and intracellular reactions of UA [26].

Both crystalline and soluble UA result in the inflammation. It is believed that UA's exposed, charged crystal surfaces enable interaction with phospholipid membranes and serum factors, contributing to the crystal-mediated inflammatory response [35]. Few other studies give us insight into inflammatory response, mediated by crystalline or soluble UA. The inflammasome-dependent and independent pathways drive crystal-mediated inflammation. It ends up with the formation of interleukin. In the case of an inflammasome-dependent route, classically the MSU crystals initiate innate immunity by activating the inflammasome routes. Consequently, interleukin-1β (IL-1β) are produced. The monocytes and the macrophages carry out such innate immune responses. On the other hand, inflammasome-independent inflammatory mechanisms are neutrophils dependent to cause the production of cytokines. Soluble urate inflammatory pathways work in the intracellular environment, where the soluble UA has numerous inflammatory and oxidative actions like intracellular immunometabolic responses, inflammatory responses, free radicals damage, and cell self-consumption [35-39].

In addition, it has been observed that hyperuricemia can independently cause endothelial dysfunction and nascent-state microalbuminuria. A nexus between urates and the amount of albumin excreted in urine was also noted. These observations were backed up by the fact that irrespective of CKD, microalbuminuria is more common in gout or hyperuricemia patients [40,41].

Yu and Cheng in their study discussed the role of UA in causing arterial stiffness and inflammation which is NLRP3 inflammasome dependent and is activated through the AMP-activated protein kinase (AMPK)-mammalian target of rapamycin (mTOR)-mitochondria-derived reactive oxygen species (mROS) and hypoxia-inducible factor 1-alpha (HIF-1α) routes. Further, they pointed out the role of hyperuricemia in seeding insulin resistance in cardiac cells via the ROS-insulin receptor substrate-1 (IRS1)/serine or threonine kinase or protein kinase B (Akt) phosphorylation route. They also noticed a strong positive correlation between hyperuricemia and the various components of MetS [25].

But Packer in his study reveals that UA is an indicator of free radicals produced by stress and is not harmful as such rather the drugs used to lower it cause the decreased production of nitric oxide, another factor that may contribute to endothelial dysfunction [29]. It is evident from the above discussion that inflammation, oxidative stress, endothelial damage, and endoplasmic reticulum stress are not only the factual pathological mechanisms that provide a cellular basis for all components of the MetS to happen but also result in the genesis of other hyperuricemia associated comorbidities.

The conventional role of anti-urate drugs in ameliorating hyperuricemia is an acknowledged and accepted fact in literature. XO inhibitors and uricosuric drugs are commonly used to treat hyperuricemia. Gherghina et al. noticed that the XO inhibitors have better results in maintaining endothelial function than uricosuric drugs in hyperuricemia patients [26]. The beneficial effects of XO inhibitors go beyond XO inhibition. Alem and some other studies reported that allopurinol's oxidoreductase inhibition improves endothelium dysfunction, downregulates the free radical damage through free radical scavenging, and reduces inflammation by decreasing NF- and TNF- formation by the human mononuclear cells in metabolic syndrome. It also lowers intercellular adhesion molecule (ICAM-1) expression and improves myeloperoxidase levels [23,42-44]. However, allopurinol use causes a substantial increase in triglyceride values after three and six months. Contrarily, febuxostat intake causes a noticeable decrease in TG levels for the initial three months but this decrease was not significant after six months [45]. Another study reported that febuxostat decreased serum cholesterol values effectively compared to allopurinol but both drugs’ performance was comparable in ameliorating LDL and HDL cholesterol [46]. Vaidya et al. observed that febuxostat decreases microalbuminuria, whereas Yu and Cheng and Suzuki et al. observed that it effectively improves cardiovascular events compared to allopurinol [25,30,31]. A review by Si et al. concluded that the main culprits of heart failure in hyperuricemia patients are XO and ROS production so they urged that XO should be the therapeutic target in such patients [28]. Likewise, Battelli et al. emphasize that pathology of the various components of MetS involves XOR which affects the regulative function of NO production, fat accumulation, ROS production, and uric acid production. The study states that the XOR inhibitors should be limited to patients with hyperuricemic effects because they play a useful role in redox equilibrium [27]. The findings of Polito et al. are also consistent with this observation [47]. This systematic review is among a few studies that attempted to investigate the cellular genesis of the MetS components and the possible role of anti-urate drugs in patients with hyperuricemia.

Study limitations

This review included studies on humans published during the past five years and only those available in English. Online libraries included only PubMed and Google Scholar databases for data collection. The study excluded non-peer-reviewed studies and research conducted on subjects below 18 years of age.

## Conclusions

This systematic review was an effort to evaluate the fundamental mechanisms that can provide the basis of the cellular genesis of MetS and its various components in hyperuricemia patients. It was also intended to find the possible effect of anti-urate drugs on MetS. We conclude that symptomatic or asymptomatic hyperuricemia is an independent risk factor for causing inflammation, oxidative stress, endothelial damage, and endoplasmic reticulum stress. These factual pathological mechanisms provide a cellular basis for all components of the MetS. They also cause the genesis of other hyperuricemia-associated comorbidities. In this context, XO inhibitors and their beneficial effects go beyond XO inhibition to ameliorate these pathological mechanisms. The precise function of hyperuricemia in oxidative stress is unclear and may be influenced by pathological and physiological circumstances. In this regard, further observational and experimental studies are needed to learn about the mechanisms responsible for the prooxidant and antioxidant events in hyperuricemia. Additionally, RCTs may help us design and develop more effective medications to treat hyperuricemia-induced pathologies including MetS and its associated comorbidities.
